# Correspondence regarding two recent publications in npj:schizophrenia about DNAm and accelerated aging in schizophrenia

**DOI:** 10.1038/s41537-017-0041-5

**Published:** 2017-10-23

**Authors:** Emilio Fernandez-Egea, Brian Kirkpatrick

**Affiliations:** 1 Cambridgeshire and Peterbourgh NHS Foundation Trust and Department of Psychiatry. Behavioural and Clinical Neuroscience Institute, University of Cambridge, Cambridge, UK; 2 Centro de Investigación Biomédica en Red de Salud Mental (CIBERSAM), Madrid, Spain; 3 Department of Psychiatry and Behavioral Sciences, Reno School of Medicine, University of Nevada, Reno, NV USA

We read with interest the recent reports by McKinney et al.^1^ and Voisey et al.^2^ Using ‘the Horvath clock’ of DNA methylation (DNAm) in prefrontal cortex^1^ and superior temporal gyrus,^2^ both studies reported no evidence of accelerated aging in the brain of people with schizophrenia compared to healthy controls.

Before offering an alternative interpretation, we would note that Horvath’s^3^ original report indicates some limitation of the DNAm technique. For instance, premature ageing diseases (progeria) such as Hutchinson–Gilford syndrome were not associated with accelerated aging using this technique, although Werner syndrome might be an exception.^4^ Additionally, the ‘clock’ is more accurate in children and young adults, which is not the population under study here. Healthy controls had a mean age in Voisey’s study of 71 years, whereas the schizophrenia group had a mean of 52 years-old. An explicit limitation is that accuracy of DNAm for predicting age strongly depends on the sample’s standard deviation of age. Both McKinney’s and Voisey’s studies only provide SEM (not SD), although age range seems large enough. Horvath also suggests an accuracy measure be reported, defined as (median) error, that is, the median absolute difference between DNAm age and chronological age. This ‘error’ is considered a measure of how well calibrated the DNAm is. None of the studies (McKinney/Voisey) provided this information. Finally, the lack of statistically difference in the age acceleration residual between schizophrenia and healthy control groups (*p* = 0.08) in Voisey’s study could be also attributed to type 2 error due to the relatively small sample size (24 per group). It would have been helpful if the authors of these two studies had provided a power calculation, given the relatively small sample sizes. We think that these methodological considerations should be taken into account when discussing the papers, and undermine one’s confidence in the conclusions.

More importantly, accelerated aging in the schizophrenia brain has been already reported.^5,6^ In order to accommodate the lack of difference in DNA methylation found are these two studies, an alternative explanation is that brain aging could be due to neural senescence. The potential of post-mitotic neurons to enter into the senescent state is increasingly recognized,^7,8^ including in schizophrenia.^9^ Senescent cells would express the aging phenotype, such as a smaller soma and dendritic loss, both of which are described in schizophrenia patients (among others in McKinney 2017, NPJ schizophrenia). Notably, Horvath’s clock is not accurate in senescent cells (see Lowe et al.^10^), suggesting that cellular aging is distinct from cellular senescence.

Taking all of the evidence together, it seems possible that people with schizophrenia may have both accelerated and cell senescence. The studies of McKinney et al. and Voisey et al. do not rule out either process.
